# Investigating the Relationship between Knowledge and Hepatotoxic Effects with Medication Adherence of TB Patients in Banyumas Regency, Indonesia

**DOI:** 10.1155/2022/4044530

**Published:** 2022-08-30

**Authors:** Dyah A. Perwitasari, Didik Setiawan, Thang Nguyen, Arum Pratiwi, Laila Rahma Fauziah, Erin Saebrinah, Triantoro Safaria, Nunuk Aries Nurulita, Idha Arfianti Wiraagni

**Affiliations:** ^1^Faculty of Pharmacy, Universitas Ahmad Dahlan, Yogyakarta 55164, Indonesia; ^2^Faculty of Pharmacy, Universitas Muhammadiyah Purwokerto, Yogyakarta 53182, Indonesia; ^3^Department of Pharmacology and Clincal Pharmacy, Can Tho University of Medicine and Pharmacy, Can Tho, Vietnam; ^4^Faculty of Psychology, Universitas Ahmad Dahlan, Yogyakarta 55164, Indonesia; ^5^Faculty of Medicine, Nursing and Public Health, Universitas Gadjah Mada, Yogyakarta 55281, Indonesia

## Abstract

Tuberculosis (TB) still remains the burden in Indonesia. One of the factors that may influence the treatment success of TB is patient's adherence. However, the hepatotoxicity of the TB medicine may decrease the patient's adherence. Our study's aim is to investigate the relationship between the patient's knowledge and the hepatotoxicity with medication adherence of TB patients in Banyumas Regency. This study was conducted at one Community Lung Health Center and two hospitals in Banyumas Regency, Purwokerto, Center of Java, Indonesia. The respondents were 91 TB patients with hepatotoxicity characterized by an increased aspartate transaminase (AST) and alanine aminotransferase (ALT). The level of the patients' knowledge about the hepatotoxicity effect was determined using a questionnaire. The patients' adherence was determined using the Medication Adherence Rating Scale -5 (MARS) questionnaire and pill count methods. Most of the patients were male (53.8%), the age was in the range of 18–29 years old (3.5%), they have no smoking history (59.3%), and their last education majorly was senior high school (46.2%). Most TB patients had poor knowledge (47.3%) and the hepatotoxic effect often appeared in grade 1 (61.5%). The TB patients with a good and moderate level of knowledge were 17.6% and 35.2%, respectively. The TB patients with moderate and severe hepatotoxicity were 39.4% and 1.1%, respectively. The measurement of the level of respondents' adherence using MARS-5 showed that 51.6% of patients had good adherence. We determined the rest of the drug-using pill count method, which resulted in 62.6% of patients adhering to taking antituberculosis drugs. TB patients with a sufficient knowledge and those with mild hepatotoxicity show the higher adherence (*p* < 0.001). There is a significant relationship between a high level of the patient's knowledge about hepatotoxicity effect, less severity of the hepatotoxic effect, and increased patient adherence in taking the medication.

## 1. Introduction

Tuberculosis (TB) is still a major problem worldwide, with around 10 million people suffering from it globally in 2018. About 44% of cases occur in Southeast Asia, with Indonesia (8%) ranking third behind India (27%) and China (9%) [1]. In 2019, there were an estimated 543,874 cases of TB in Indonesia, with West Java, East Java, and Central Java, as the areas with the highest number of cases, having 99,398, 56,445, and 67,063 cases, respectively [2]. Indonesia was in the third rank of the highest TB burden countries in the world, with the incidence reached 8.4%, after India (26%) and China (8.5%) [3]. The TB death case in Indonesia was 15.186 in 2021 and the estimation on TB cases was 824.000 [4]. Meaning while, the new cases of TB in center of Java in 2020 reach 118 per 100.000 population [5].

The presence of hepatotoxic side effects and the lack of knowledge on TB and its treatment can cause worsening of the disease since it affects patient's adherence. Patients with a high level of knowledge are more likely to adhere to treatment than those without it [6]. Some factors may affect the adherence of TB patients, like structural factors, personal factors, social factors, and health services factors. Side effects, directly observed treatment short course (DOTs) availability, and duration of treatment are parts of the structural factors. Personal factors include poverty and gender, and stigma is part of the social factor. The other patients' factors, such as knowledge and attitude, also may influence patients' interactions with health services [7,8]. Some interventions to increase adherence to TB patients include education, counseling, psychological intervention, reminders, and tracers [9]. Since 2018, the World Health Organization published a handbook to increase the TB patients' adherence to the medication. The handbook explained about digital technologies which will be used to support the medication adherence [10]. In Indonesia, the TOSS Program (“Temukan dan Obati Sampai Sembuh TBC”: Find and treat the TB patients) has been launched since 2019 to increase the TB patients' adherence in taking the medicine [11].

Meanwhile, hepatotoxic side effects in TB patients are usually caused by using antituberculosis drugs, which can lead to patients discontinuing treatment since a high severity of the adverse effects of oral antituberculosis will lead to decreased adherence to taking the medication [12]. The newest study conducted in Ethiopia also showed that the hepatotoxicity caused by antituberculosis reached 7.9%, with a predictive diagnosis of extrapulmonary TB, having comorbid and old age [13]. The incidence of hepatotoxicity in Indonesia resulting from the use of antituberculosis drugs is about 50% with early markers, such as increased levels of aspartate amino transaminase/glutamate oxaloacetate transaminase (AST/GOT) and/or alanine amino transferase/glutamate pyruvate transaminase (ALT/GPT) [14].

Factors that can lead to a rise in TB must be prevented so that cases can be decreased. Therefore, this study investigates the relationship between the patient's level of knowledge and the hepatotoxic effect with medication adherence of TB patients in the Banyumas Regency, Purwokerto.

## 2. Materials and Methods

### 2.1. Study Design

This research used observational analysis with a cross-sectional approach on patients with pulmonary TB who met the inclusion and exclusion criteria at the Community Lung Health Center and two hospitals in Banyumas Regency, Purwokerto, between August 2020–January 2021. Banyumas is a rural area, with most of the population work as the farmers. Most of the area of this regency are plantation and rice fields. The new cases of TB in this area was 178 per 100.000 population, compared to the new cases of Indonesia, which was 300 per 100.000 population [4,15].

The study's inclusion criteria were pulmonary TB adults' patients currently undergoing treatment, over 15 years, willing to be a respondent, had high AST and ALT examination results, and living in the city, rural and urban area of Purwokerto.

Meanwhile, the exclusion criteria included new patients, that died, did not answer the questionnaire, stopped treatment, or were referred to other hospitals, and TB patients with complications such as gout, diabetes mellitus, liver disorders, and HIV.

**Figure 1:**
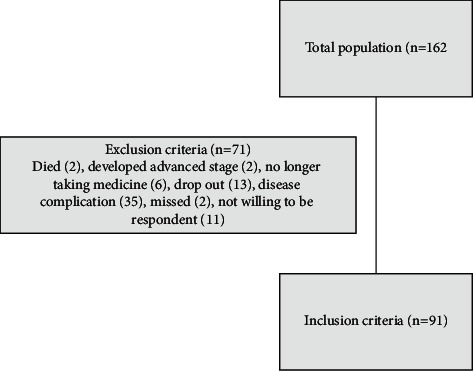
Flowchart of subjects' recruitment.

We did not define the study sample size, due to the pandemic situation. During the pandemic situation, the number of TB patients who visited the healthcare centers was decrease; thus, we recruited the all patients visited the healthcare centers.  Figure 1 presents the flowchart of subjects' recruitment.

### 2.2. Instruments

The patient's knowledge and adherence were obtained through direct interviews with respondents using knowledge and adherence questionnaires. The knowledge questionnaire contains 21 questions consisting of 13 questions regarding TB treatment and 8 questions about oral antituberculosis side effects. The questions in the questionnaire were favorable and we categorized the patient' level of knowledge into poor, moderate, and high, based on the right answer. The proportion of right answer more than 80%, 60%–79%, and below 50% were categorized into high, moderate, and poor, respectively. Meanwhile, the adherence questionnaire used the medication adherence rating scale (MARS), which consisted of 5 questions with a frequency scale of 1 to 5 (always, often, sometimes, rarely, and never) and the calculation of the remaining medication (pill count).

### 2.3. Data Analysis

Statistical analysis was performed using SPSS 16.0 for Windows (SPSS Inc. Chicago, IL, United States). The statistical analysis in this study was performed using univariate and bivariate analyses. The characteristic data are presented in the table. The relationship between the level of knowledge, hepatotoxicity, and patient adherence was assessed using the chi-square statistical test. The *p* values less than 0.05 were considered to indicate a statistical significance.

## 3. Results

In general, our study finds significant relationships between patients' knowledge and hepatotoxic effect with adherence. TB patients with higher knowledge have better adherence and TB patients with mild hepatotoxic effects are related to higher adherence.

The study subjects included 162 respondents. Of the total population, 11 were unwilling to be respondents and 35 were patients with complications. Therefore, only 91 respondents met the inclusion criteria.

**Figure 2:**
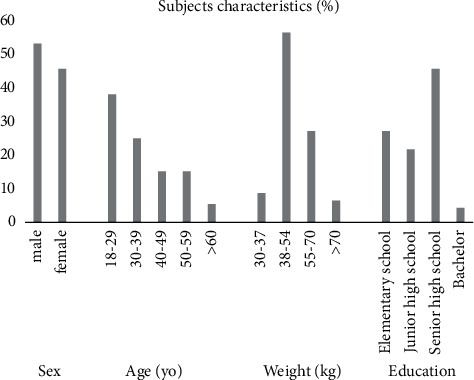
Subjects' characteristics.

**Figure 3:**
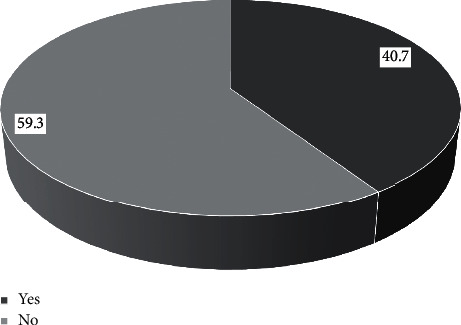
Smoking history.

**Figure 4:**
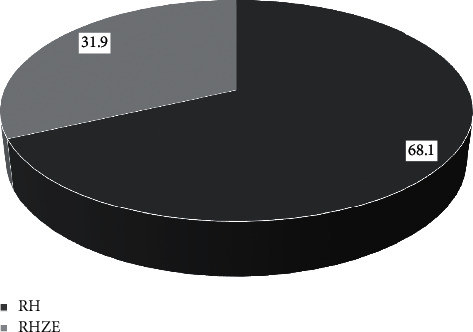
Proportion of antituberculosis. RH: rifampicin and isoniazid; RHZE: rifampicin, isoniazid, pyrazinamide, and ethambutol.

A total of 91 TB patients met the inclusion criteria and were in the final analysis. Most of them were male (53.8%), aged around 18–29 years (38.5%), and bodyweight of 38–54 kg (57.1%). In this study, most TB patients had no smoking history (59.3%). The patient's last education level is high, namely, senior high school (46.2%) and is in the advanced treatment phase by taking the antituberculosis drug rifampin and isoniazid (68.1%) (Figures 2–4).

The outcomes of this study were hepatotoxic effects (AST and ALT), level of patient's knowledge, and the score of patient's adherences. The patient's knowledge and the score of patient's adherences were the predictors of the patient's adherence. All the TB patients were taking the TB medicines as the exposure. The potential confounder of this study was the role of social workers. The AST and ALT were collected from the patient's medical record. According to liver tox sources (2019), there are 4 stages of hepatotoxicity, namely, stage 1 (mild = AST/ALT 51–125 U/L), stage 2 (moderate = AST/ALT 126–250 U/L), stage 3 (severe = AST/ALT 251–500 U/L), and stage 4 (life-threatening = AST/ALT > 500 U/L) [16].

**Figure 5:**
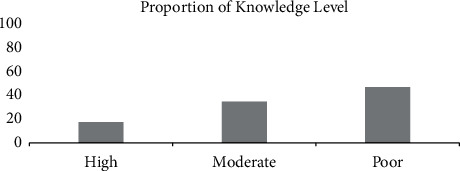
Proportion of the subjects' knowledge level.

**Figure 6:**
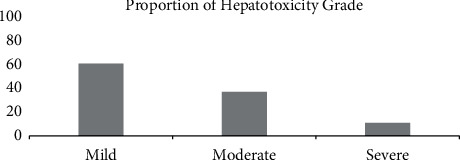
Proportion of the subjects' hepatotoxicity grade.

**Figure 7:**
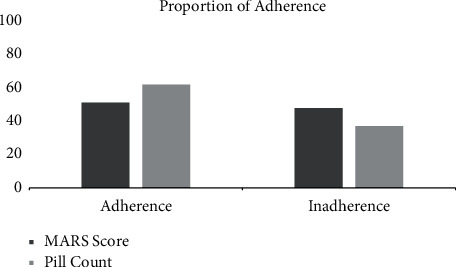
Proportion of subjects' adherence based on MARS and pill count.

Most TB patients had poor knowledge (47.3%) (Figure 5) and the hepatotoxic effect often appeared in grade 1 (61.5%) (Figure 6). Measurement of the level of respondents' adherence using MARS-5 showed that 51.6% of patients had good adherence. We determined the rest of the drug-using pill count method, which resulted in 62.6% of patients adhering to taking antituberculosis drugs (Figure 7).

The significant relationship can be seen in Table 1, between the level of knowledge, hepatotoxicity, and the patient's adherence.

## 4. Discussion

Our study finds the significant association between knowledge-adherence and hepatotoxicity effect-adherence. The TB patients in our study did not have any comorbidities which can cause the hepatotoxicity effect during the TB treatment. The previous studies found that some characteristic factors were not related to the hepatotoxic effect. The age, gender alcohol intake, and body mass index (BMI) did not have any relationship with hepatotoxic effects [17,18]. Another study explained that women might be at risk for TB because a patriarchal culture is common in Indonesian society; therefore, men have better health than women. Based on the facts in developing countries, most low-income families are exposed to smoke from firewood or biogas, which is burned as fuel, causing an increase in the incidence of TB in poor women in developing countries [18]. Furthermore, because many poor women live in homes with poor lighting and ventilation, the risk of being infected with TB bacteria is higher [18]. In a particular community, males were more aware of TB than females [19].

TB is more at risk of being transmitted in the productive age group (15–55 years) because at this age, humans are physically and biologically mature and are in dense activity and do more activities outside the home such as working and socializing [20]. Our study findings align with the previous studies, which also found that most TB patients were in productive age [21,22]. Therefore, they often forget to visit for treatment and take medication regularly. According to another study, TB is also a disease that can attack all ages, both adults and productive ages. It can happen because the level of activity and work at that age can make it easy to get infected by other TB sufferers [21].

TB patients usually have low body weight or are thin (38–54 kg) and often malnutrition [20]. It will aggravate the infectious disease, making nutritional status the major cause of treatment failure in TB patients. Furthermore, malnutrition and TB are two interconnected problems in which malnutrition can impact immune system deterioration and make the affected people's immune systems more susceptible to TB infection than healthy people's immune systems [18,23]. TB is often interconnected with poverty, malnutrition, hygiene, overcrowding, and lack of knowledge [20]. Nausea and vomiting are the symptoms of hepatotoxic effects. TB patients who experienced these symptoms may experience the loss of appetite, then may influence the number of nutrition needed [24,25]. The previous study mentioned that nausea/vomiting was highly prevalent then may cause the non-adherence of dietary counseling [24].

A systematic review and meta-analysis about interventions provided to the TB patients to increase their adherence showed that patient education and counseling was one of the interventions needed [9]. Thus, the level of education may influence patients' knowledge and attitude during TB treatment. The previous study in Karachi also found that the educated patients were more helpful in changing their lifestyle, including their awareness in preventing the spread of the disease. The more literate patients, the more effort to improve their health [26]. A survey conducted to the community in Pakistan showed that people with higher education had more concern about the prevalence and threats of the disease [19].

Cigarette smoke plays a role in the pathogenesis of TB. For smokers, nicotine will activate the nicotinic receptor and will reduce the production of TNF-*α*. This mechanism leads to a decrease in protection and causes more susceptibility to TB [27]. There was a prediction that between 2010 and 2050, the new cases of TB were caused by smoking history. It is also predicted that smoking will increase the number of TB-related death. Both active and passive smokers were associated with the risk of TB. The history of parental smoking is also associated with respiratory infection in children. Thus, the education for active smokers that they can cause harm to their environment, especially related to TB, must be conducted [28].

Treatment of TB can be divided into two phases: the intense phase, which lasts two months, and the continuation phase, which lasts four to seven months. Two studies presented that hepatotoxicity can be found during the initial phase [12,29]. However, a study reported that hepatotoxicity occurred after the 12 weeks of treatment initiation with the addition of pyrazinamide [30].

Our study results are also in line with the previous studies, which presented that better knowledge about TB is related to the patients' adherence [31,32]. The previous qualitative study in Jepara, Indonesia, also stated that knowledge about disease and treatment; also, the healthcare facilities influenced the patients adherence [33]. The TB patients who experienced mild hepatotoxicity had a significant association with the good adherence; however, TB patients who experienced mild and severe hepatotoxicity had significant association with low adherence. The side effects experienced by TB patients could influence their adherence. The long duration of treatment may cause adverse event that greatly influence the adherence. The unpleasant effect may cause fear for the patients; then, they will stop the treatment [32,34]. Thus, education about the side effects and treatment of the side effects will increase patients' adherence. Even though the patients' adherence is good in some studies, health education is still needed to eliminate factors that may influence the adherence and treatment success rate. Other factors, like the cost of transportation, lack of awareness, and distance, also have to be considered in increasing the patients' adherence [35,36]. This study finding will support the government to provide additional care for TB patients, which include the education and side effect during the TB treatment.

We present the association between knowledge level, hepatotoxicity effect, and the patient's adherence as the strength of this study. The limited sample size became the limitation of our study because we conducted our study in a pandemic situation. During the pandemic, TB patients also felt insecure about visiting the hospitals. We did not involve the role of the social workers in this study, because we want to define the patients' knowledge and adherence, which are correlated with the hepatotoxicity effect.

## 5. Conclusions

The results showed that most TB patients had a low knowledge level, a mild hepatotoxic level (stage 1), and a high adherence level. The patients' knowledge about hepatotoxic effects and hepatotoxic effects are the significant predictors of adherence. Our study can be the pilot study for the government. The education for the TB patients about the treatment outcome and side effects of TB can be the important steps to increase the patients' adherence. The long duration of TB treatment may result the side effect that could deteriorate the patients' adherence. Thus, the massive education and side effect monitoring program can be the solution of treatment non-adherence. Since TB is still becoming the burden in Indonesia and Indonesia is in the second top rank of TB burden country, the massive program of education and side effect monitoring, hopefully, will decrease the burden. This program also can be scaled up in other top ranks TB countries, such as China, India, and South Africa.

## Figures and Tables

**Table 1:** Relationship between knowledge level and hepatotoxic effect with patient adherence.

	*Adherence*		Total (91)	*p* Value
	Low (%)	High (%)		
*Knowledge level*				
High (>75%)	2 (12.5%)	14 (87.5%)	16	<0.001
Moderate (60-74%)	6 (18.8%)	26 (81.2%)	32	
Poor (<60%)	36 (83.7%)	7 (16.3%)	43	

*Hepatotoxic Effect*				
*Grade* 1 *(Mild)* (AST/ALT 51–125 U/L)	13 (23.3%)	43 (76.8%)	56	<0.001
*Grade* 2 (M*oderate*) (AST/ALT 126–250 U/L)	30 (88.2%)	4 (11.8%)	34	
*Grade* 3 (S*evere*) (AST/ALT 251–500 U/L)	1 (100%)	0 (0%)	1	

## Data Availability

If the readers are interesting to get the data, the readers can contact corresponding author (Dyah Aryani Perwitasari: diahperwitasari2003@yahoo.com). We are still developing the data base for tuberculosis in Indonesia; thus, we need to secure the data.

## Supplementary Materials

Supplementary Materials The supplementary data are questionnaire form and the data sheet of this study.
