# Supplementary material for: Investigating the Relationship between Knowledge and Hepatotoxic Effects with Medication Adherence of TB Patients in Banyumas Regency, Indonesia
Source: Int J Clin Pract. 2022 Aug 30;2022:4044530. doi: 10.1155/2022/4044530 (PMC9448620; doi:10.1155/2022/4044530)
Supplement: Supplementary Materials — The supplementary data are questionnaire form and the data sheet of this study. [file 4044530.f1.zip › Supplementary materials Eng.pdf]

| Code | Age | Sex | BW | AST | ALT | Education | % Knowledge | Category | TOTAL MARS-5 | MARS category | Pill Count | Adherence category | Smoking | Medication |
|---|---|---|---|---|---|---|---|---|---|---|---|---|---|---|
| R1 | 24 | 1 | 53 | 59 | 102 | SHS | 80,95% | Good | 25 | Comply | 100% | Adherent | No | RH |
| R2 | 32 | 2 | 38 | 193 | 180 | ES | 57,14% | Bad | 23 | Not Comply | 78,50% | Non-adherent | No | RH |
| R3 | 20 | 2 | 65 | 74 | 73 | SHS | 76,19% | Good | 20 | Not Comply | 71,42% | Non-adherent | No | RH |
| R4 | 31 | 1 | 57 | 51 | 68 | SHS | 76,19% | Good | 25 | Comply | 100% | Adherent | Yes | RH |
| R5 | 58 | 1 | 57 | 202 | 186 | ES | 61,90% | Enough | 25 | Comply | 100% | Adherent | Yes | RH |
| R6 | 71 | 2 | 58 | 130 | 136 | ES | 47,61% | Bad | 24 | Not Comply | 79% | Non-adherent | No | RH |
| R7 | 48 | 2 | 38 | 131 | 134 | ES | 52,38% | Bad | 20 | Not Comply | 50% | Non-adherent | No | RHZE |
| R8 | 37 | 1 | 57 | 137 | 125 | ES | 71,42% | Enough | 24 | Not Comply | 78,50% | Non-adherent | Yes | RHZE |
| R9 | 19 | 2 | 50 | 56 | 74 | SHS | 80,95% | Good | 22 | Not Comply | 50% | Non-adherent | No | RH |
| R10 | 30 | 2 | 55 | 56 | 54 | ES | 52,38% | Bad | 24 | Not Comply | 78,50% | Non-adherent | No | RH |
| R11 | 40 | 1 | 58 | 247 | 168 | YHS | 52,38% | Bad | 25 | Comply | 100% | Adherent | Yes | RH |
| R12 | 34 | 2 | 60 | 56 | 90 | SHS | 80,95% | Good | 25 | Comply | 100% | Adherent | No | RH |
| R13 | 23 | 2 | 54 | 53 | 80 | SHS | 85,71% | Good | 25 | Comply | 100% | Adherent | No | RH |
| R14 | 37 | 1 | 52 | 53 | 80 | YHS | 52,38% | Bad | 25 | Comply | 100% | Adherent | Yes | RH |
| R15 | 33 | 2 | 55 | 63 | 57 | YHS | 52,38% | Bad | 23 | Not Comply | 78,50% | Non-adherent | Yes | RH |
| R16 | 39 | 1 | 49 | 131 | 215 | ES | 57,14% | Bad | 23 | Not Comply | 78,50% | Non-adherent | Yes | RH |
| R17 | 41 | 1 | 36 | 53 | 56 | ES | 47,61% | Bad | 25 | Not Comply | 71% | Non-adherent | Yes | RH |
| R18 | 38 | 2 | 45 | 67 | 52 | SHS | 71,42% | Enough | 25 | Comply | 100% | Adherent | No | RH |
| R19 | 27 | 2 | 54,5 | 51 | 51 | Bachelor | 80,95% | Good | 25 | Comply | 100% | Adherent | No | RHZE |
| R20 | 22 | 2 | 41 | 145 | 134 | ES | 57,14% | Bad | 24 | Not Comply | 78,50% | Non-adherent | No | RHZE |
| R21 | 44 | 2 | 45 | 196 | 189 | ES | 66,67% | Enough | 23 | Not Comply | 76% | Non-adherent | No | RH |
| R22 | 54 | 2 | 56 | 171 | 134 | ES | 57,14% | Bad | 24 | Not Comply | 71% | Non-adherent | No | RH |
| R23 | 20 | 2 | 51 | 56 | 62 | SHS | 66,67% | Enough | 25 | Comply | 100% | Adherent | No | RH |
| R24 | 50 | 2 | 50 | 144 | 150 | SHS | 80,95% | Good | 25 | Comply | 100% | Adherent | No | RH |
| R25 | 47 | 2 | 76 | 234 | 135 | ES | 52,38% | Bad | 24 | Not Comply | 100% | Adherent | No | RH |
| R26 | 20 | 1 | 54 | 130 | 133 | SHS | 61,90% | Enough | 23 | Not Comply | 71,40% | Non-adherent | No | RH |
| R27 | 48 | 2 | 52 | 71 | 70 | ES | 61,90% | Enough | 23 | Not Comply | 76% | Non-adherent | No | RH |
| R28 | 54 | 1 | 65 | 81 | 50 | SHS | 61,67% | Enough | 21 | Comply | 100% | Adherent | Yes | RH |

| Code | Age | Sex | BW | AST | ALT | Education | % Knowledge | Category | TOTAL MARS-5 | MARS category | Pill Count | Adherence category | Smoking | Medication |
|---|---|---|---|---|---|---|---|---|---|---|---|---|---|---|
| R29 | 27 | 1 | 65 | 133 | 136 | SHS | 71,42% | Enough | 24 | Not Comply | 67,85% | Non-adherent | Yes | RH |
| R30 | 54 | 1 | 58 | 206 | 231 | ES | 57,14% | Bad | 24 | Not Comply | 79% | Non-adherent | Yes | RH |
| R31 | 50 | 1 | 50 | 67 | 66 | YHS | 61.90 | Enough | 25 | Comply | 100% | Adherent | Yes | RHZE |
| R32 | 39 | 2 | 58 | 78 | 66 | SHS | 61.90 | Enough | 25 | Comply | 100% | Adherent | No | RHZE |
| R33 | 42 | 2 | 50 | 79 | 94 | YHS | 57.14 | Bad | 25 | Comply | 100% | Adherent | No | RHZE |
| R34 | 24 | 2 | 47 | 191 | 169 | SHS | 52.38 | Bad | 21 | Not Comply | 66.67% | Non-adherent | No | RHZE |
| R35 | 52 | 1 | 49 | 132 | 127 | YHS | 57.14 | Bad | 21 | Not Comply | 58.33% | Non-adherent | Yes | RH |
| R36 | 40 | 2 | 40 | 183 | 128 | SHS | 47.62 | Bad | 19 | Not Comply | 63.06% | Non-adherent | Yes | RHZE |
| R37 | 70 | 1 | 40 | 299 | 402 | ES | 47.62 | Bad | 17 | Not Comply | 16.67% | Non-adherent | No | RHZE |
| R38 | 31 | 2 | 50 | 76 | 62 | YHS | 61.90 | Enough | 25 | Comply | 100% | Adherent | No | RHZE |
| R39 | 29 | 1 | 45 | 59 | 85 | SHS | 61.90 | Enough | 25 | Comply | 100% | Adherent | Yes | RH |
| R40 | 25 | 2 | 35 | 92 | 79 | SHS | 57.14 | Bad | 25 | Comply | 100% | Adherent | No | RHZE |
| R41 | 29 | 2 | 50 | 84 | 75 | SHS | 52.38 | Bad | 21 | Not Comply | 90.47% | Adherent | No | RHZE |
| R42 | 57 | 1 | 45 | 160 | 144 | ES | 52.38 | Bad | 21 | Not Comply | 76.19% | Non-adherent | No | RHZE |
| R43 | 22 | 2 | 44 | 160 | 151 | SHS | 47.62 | Bad | 19 | Not Comply | 64.28% | Non-adherent | No | RHZE |
| R44 | 25 | 1 | 45 | 133 | 129 | SHS | 47.62 | Bad | 21 | Not Comply | 76.19% | Non-adherent | Yes | RHZE |
| R45 | 21 | 2 | 44 | 145 | 135 | SHS | 52.38 | Bad | 19 | Not Comply | 16.67% | Non-adherent | No | RH |
| R46 | 36 | 1 | 45 | 133 | 129 | YHS | 52.38 | Bad | 19 | Not Comply | 78.57% | Non-adherent | Yes | RHZE |
| R47 | 36 | 1 | 46 | 89 | 90 | SHS | 57.14 | Bad | 21 | Not Comply | 85.71% | Adherent | Yes | RHZE |
| R48 | 23 | 2 | 74 | 88 | 78 | SHS | 57.14 | Bad | 23 | Not Comply | 92.86% | Adherent | No | RHZE |
| R49 | 24 | 2 | 35 | 77 | 76 | SHS | 52.38 | Bad | 23 | Not Comply | 78.57% | Non-adherent | No | RHZE |
| R50 | 50 | 2 | 63 | 181 | 167 | ES | 47.62 | Bad | 19 | Not Comply | 78.57% | Non-adherent | No | RHZE |
| R51 | 57 | 1 | 35 | 149 | 150 | ES | 47.62 | Bad | 21 | Not Comply | 85.71% | Adherent | No | RHZE |
| R52 | 21 | 2 | 46 | 76 | 63 | SHS | 61.90 | Enough | 25 | Comply | 100% | Adherent | No | RH |
| R53 | 47 | 1 | 52 | 188 | 192 | YHS | 52.38 | Bad | 21 | Not Comply | 58.33% | Non-adherent | No | RHZE |
| R54 | 31 | 1 | 37 | 77 | 60 | SHS | 61.90 | Enough | 25 | Comply | 100% | Adherent | Yes | RHZE |
| R55 | 46 | 1 | 44 | 180 | 194 | YHS | 47.62 | Bad | 21 | Not Comply | 78.57% | Non-adherent | No | RHZE |
| R56 | 21 | 1 | 44 | 95 | 70 | SHS | 47.62 | Bad | 23 | Not Comply | 78.57% | Non-adherent | No | RHZE |

| Code | Age | Sex | BW | AST | ALT | Education | % Knowledge | Category | TOTAL MARS-5 | MARS category | Pill Count | Adherence category | Smoking | Medication |
|---|---|---|---|---|---|---|---|---|---|---|---|---|---|---|
| R57 | 38 | 1 | 85 | 184 | 194 | YHS | 52.38 | Bad | 23 | Not Comply | 16.67% | Non-adherent | Yes | RH |
| R58 | 20 | 2 | 37 | 145 | 159 | YHS | 47.62 | Bad | 19 | Not Comply | 16.67% | Non-adherent | No | RH |
| R59 | 25 | 1 | 42 | 197 | 166 | SHS | 47.62 | Bad | 21 | Not Comply | 50% | Non-adherent | Yes | RH |
| R60 | 69 | 1 | 49 | 66 | 84 | ES | 61.90 | Enough | 25 | Comply | 100% | Adherent | No | RHZE |
| R61 | 34 | 1 | 56 | 22 | 53 | SHS | 61,90 | Enough | 25 | Comply | 100% | Adherent | Yes | RH |
| R62 | 27 | 2 | 57 | 43 | 39 | SHS | 66,67 | Enough | 25 | Comply | 100% | Adherent | No | RH |
| R63 | 58 | 1 | 45 | 44 | 41 | ES | 52,38 | Bad | 25 | Comply | 90% | Adherent | Yes | RH |
| R64 | 37 | 1 | 52,5 | 43 | 136 | SHS | 57,14 | Bad | 23 | Not Comply | 90% | Adherent | Yes | RH |
| R65 | 26 | 2 | 50 | 31 | 17 | SHS | 76,19 | Good | 25 | Comply | 100% | Adherent | No | RH |
| R66 | 21 | 1 | 45 | 35 | 22 | SHS | 61,90 | Enough | 24 | Not Comply | 100% | Adherent | Yes | RH |
| R67 | 30 | 1 | 46,5 | 38 | 36 | SHS | 66,67 | Enough | 25 | Comply | 100% | Adherent | Yes | RH |
| R68 | 18 | 1 | 53,5 | 58 | 148 | YHS | 57,14 | Bad | 24 | Not Comply | 100% | Adherent | No | RH |
| R69 | 37 | 1 | 97 | 36 | 39 | Bachelor | 85,71 | Good | 25 | Comply | 100% | Adherent | Yes | RH |
| R70 | 65 | 2 | 38 | 32 | 25 | ES | 66,67 | Enough | 25 | Comply | 100% | Adherent | No | RH |
| R71 | 29 | 2 | 44,5 | 47 | 38 | SHS | 76,19 | Good | 25 | Comply | 100% | Adherent | No | RH |
| R72 | 25 | 1 | 68 | 39 | 34 | Bachelor | 80,95 | Good | 25 | Comply | 100% | Adherent | No | RH |
| R73 | 40 | 1 | 47 | 44 | 30 | ES | 61,90 | Enough | 25 | Comply | 100% | Adherent | Yes | RHZE |
| R74 | 25 | 1 | 55 | 40 | 19 | SHS | 66,67 | Enough | 25 | Comply | 100% | Adherent | Yes | RHZE |
| R75 | 39 | 2 | 40 | 33 | 18 | YHS | 71,43 | Enough | 25 | Comply | 100% | Adherent | No | RH |
| R76 | 19 | 1 | 59 | 35 | 10 | SHS | 61,90 | Enough | 25 | Comply | 100% | Adherent | No | RH |
| R77 | 53 | 1 | 42 | 69 | 40 | YHS | 76,19 | Good | 25 | Comply | 100% | Adherent | Yes | RH |
| R78 | 47 | 2 | 36 | 132 | 37 | ES | 57,14 | Bad | 25 | Not Comply | 100% | Adherent | No | RH |
| R79 | 35 | 2 | 53 | 55 | 24 | YHS | 57,14 | Bad | 25 | Comply | 100% | Adherent | No | RH |
| R80 | 31 | 1 | 90 | 80 | 50 | Bachelor | 76,19 | Good | 25 | Comply | 90% | Adherent | No | RH |
| R81 | 47 | 1 | 56 | 33 | 45 | YHS | 66,67 | Enough | 25 | Comply | 100% | Adherent | Yes | RH |
| R82 | 21 | 1 | 62 | 46 | 85 | YHS | 61,90 | Enough | 25 | Comply | 100% | Adherent | Yes | RH |
| R83 | 20 | 1 | 58,5 | 42 | 56 | YHS | 57,14 | Bad | 24 | Not Comply | 85% | Adherent | No | RH |

| Code | Age | Sex | BW | AST | ALT | Education | % Knowledge | Category | TOTAL MARS-5 | MARS category | Pill Count | Adherence category | Smoking | Medication |
|---|---|---|---|---|---|---|---|---|---|---|---|---|---|---|
| R84 | 56 | 1 | 75,5 | 45 | 48 | SHS | 66,67 | Enough | 25 | Comply | 100% | Adherent | Yes | RH |
| R85 | 50 | 2 | 30 | 38 | 16 | ES | 66,67 | Enough | 25 | Comply | 100% | Adherent | No | RH |
| R86 | 38 | 2 | 45,5 | 43 | 38 | SHS | 66,67 | Enough | 25 | Comply | 100% | Adherent | No | RH |
| R87 | 71 | 1 | 43 | 38 | 51 | ES | 47,62 | Bad | 25 | Comply | 100% | Adherent | Yes | RH |
| R88 | 21 | 2 | 46 | 35 | 16 | SHS | 80,95 | Good | 25 | Comply | 100% | Adherent | No | RH |
| R89 | 21 | 1 | 54 | 43 | 55 | SHS | 76,19 | Good | 25 | Comply | 100% | Adherent | Yes | RH |
| R90 | 41 | 1 | 65 | 56 | 66 | SHS | 71,43 | Enough | 25 | Comply | 100% | Adherent | Yes | RH |
| R91 | 26 | 1 | 43,5 | 108 | 84 | YHS | 61,90 | Enough | 25 | Comply | 100% | Adherent | Yes | RH |

Pendidikan:

SMA: SHS;

SMP: YHS;

SD: ES;

S1: Bachelor

Category

Baik: Good

Cukup: enough

Kurang: Bad

MARS category

Tinggi : comply

Rendah:  not comply

Smoking

Ya: Yes

Tidak : No

Questionnaire

**FORM 2**

# Data Report Form

## A. Patient Data

| | |
|---|---|
| Initial | : |
| Treatment; month | 1 2 3 4 5 6 …….. |
| Age<br>Body weight | : |
| Sex | : |
| Address | : |
| Mobile phone number | : |
| Last education | : |
| Work | : |
| Salary/month | : |
| Diagnosis | : |
| Complication | : |
| Comorbidity | : |
| Medication | : |

B. **Patient adherence (MARS)**

Choose the statement which appropriate to your condition (V)

| Statement | | | | | |
|---|---|---|---|---|---|
| | **Always** | **Frequently** | **sometimes** | **Rare** | **Never** |
| I forgot taking the drug | | | | | |
| I change the dose of the drug | | | | | |
| I stop the drug for a while | | | | | |
| I decide to take the minimum dose of the drug | | | | | |

C. Knowledge about side effect

Choose the answer which is appropriate to your condition

1. **Do you know that the drugs will cure the tuberculosis?**

This drug will prevent the relapse Yes ☐ No ☐

I don't know Yes ☐ No ☐

Other:……………………………………………………………………………………

2. **How many TB drugs which you take in one day?**

4 ☐

3 plus vitamine ☐

3 ☐

I don't know ☐

Others……………………………………………………………………………………

Name of the drugs.......................................................................

3. **How many times do you take TB drugs in a day?**

1 x ☐

2 x ☐

3 x ☐

I don’t know ☐

Others………………………………………………………………………………………….

4. **How long do ou take the TB drugs?**

More than 9 months Yes ☐ No ☐

Less than 6 months Yes ☐ No ☐

6-9 months Yes ☐ No ☐

I don’t know Yes ☐ No ☐

Others………………………………………………………………………………………….

5. **Do you know the side effect of TB drugs?**

hepatotoxicity Yes ☐ No ☐

nausea-vomiting Yes ☐ No ☐

stomachache Yes ☐ No ☐

jaundice Yes ☐ No ☐

neuropaty Yes ☐ No ☐

burn in foot Yes ☐ No ☐

fever Yes ☐ No ☐

Rash Yes ☐ No ☐

Others…………………………………………………….

6. **What should you do when you experience TB drugs side effect?**

Stop taking the drug Yes ☐ No ☐

Consultation to physician Yes ☐ No ☐

Laboratory check Yes ☐ No ☐

I don't know Yes ☐ No ☐

Others…………………………………………………………………………………….

7. Did you experience these side effect, during the TB treatment?

hepatotoxicity Yes ☐ No ☐

nausea-vomiting Yes ☐ No ☐

stomachache Yes ☐ No ☐

jaundice Yes ☐ No ☐

neuropaty Yes ☐ No ☐

burn in foot Yes ☐ No ☐

fever Yes ☐ No ☐

Rash Yes ☐ No ☐

Others…………………………………………………….

*Thank you for your paticipating in this research.*

*Hopefully your condition gets better soon.*
