# Supplementary material for: Investigating the Relationship between Knowledge and Hepatotoxic Effects with Medication Adherence of TB Patients in Banyumas Regency, Indonesia
Source: Int J Clin Pract. 2022 Aug 30;2022:4044530. doi: 10.1155/2022/4044530 (PMC9448620; doi:10.1155/2022/4044530)
Supplement: Supplementary Materials — The supplementary data are questionnaire form and the data sheet of this study. [file 4044530.f1.zip › Supplementary materials.pdf]

Kode	Umur	JK	BB	AST	ALT	Kategori	Pendidikan	% Pengetahuan	KATEGORI	TOTAL MARS-5	KAT MARS	Pill Count	KATEGORI	MEROKOK	Obat
R1	24	1	53	59	102	1	SMA	80,95%	Baik	25	Tinggi	100%	Patuh	Tidak	RH
R2	32	2	38	193	180	2	SD	57,14%	Kurang	23	Rendah	78,50%	Tidak patuh	Tidak	RH
R3	20	2	65	74	73	1	SMA	76,19%	Baik	20	Rendah	71,42%	Tidak patuh	Tidak	RH
R4	31	1	57	51	68	1	SMA	76,19%	Baik	25	Tinggi	100,00%	Patuh	Ya	RH
R5	58	1	57	202	186	2	SD	61,90%	Cukup	25	Tinggi	100%	Patuh	Ya	RH
R6	71	2	58	130	136	2	SD	47,61%	Kurang	24	Rendah	79%	Tidak patuh	Tidak	RH
R7	48	2	38	131	134	2	SD	52,38%	Kurang	20	Rendah	50%	Tidak patuh	Tidak	RHZE
R8	37	1	57	137	125	2	SD	71,42%	Cukup	24	Rendah	78,50%	Tidak patuh	Ya	RHZE
R9	19	2	50	56	74	1	SMA	80,95%	Baik	22	Rendah	50%	Tidak patuh	Tidak	RH
R10	30	2	55	56	54	1	SD	52,38%	Kurang	24	Rendah	78,50%	Tidak patuh	Tidak	RH
R11	40	1	58	247	168	2	SMP	52,38%	Kurang	25	Tinggi	100,00%	Patuh	Ya	RH
R12	34	2	60	56	90	1	SMA	80,95%	Baik	25	Tinggi	100%	Patuh	Tidak	RH
R13	23	2	54	53	80	1	SMA	85,71%	Baik	25	Tinggi	100%	Patuh	Tidak	RH
R14	37	1	52	53	80	1	SMP	52,38%	Kurang	25	Tinggi	100%	Patuh	Ya	RH
R15	33	2	55	63	57	1	SMP	52,38%	Kurang	23	Rendah	78,50%	Tidak patuh	Ya	RH
R16	39	1	49	131	215	2	SD	57,14%	Kurang	23	Rendah	78,50%	Tidak patuh	Ya	RH
R17	41	1	36	53	56	1	SD	47,61%	Kurang	25	Rendah	71%	Tidak patuh	Ya	RH
R18	38	2	45	67	52	1	SMA	71,42%	Cukup	25	Tinggi	100%	Patuh	Tidak	RH
R19	27	2	54,5	51	51	1	S1	80,95%	Baik	25	Tinggi	100,00%	Patuh	Tidak	RHZE

R20	22	2	41	145	134	2	SD	57,14%	Kurang	24	Rendah	78,50%	Tidak patuh	Tidak	RHZE	
R21	44	2	45	196	189	2	SD	66,67%	Cukup	23	Rendah	76%	Tidak patuh	Tidak	RH	
R22	54	2	56	171	134	2	SD	57,14%	Kurang	24	Rendah	71%	Tidak patuh	Tidak	RH	
R23	20	2	51	56	62	1	SMA	66,67%	Cukup	25	Tinggi	100%	Patuh	Tidak	RH	
R24	50	2	50	144	150	2	SMA	80,95%	Baik	25	Tinggi	100%	Patuh	Tidak	RH	
R25	47	2	76	234	135	2	SD	52,38%	Kurang	24	Rendah	100%	Patuh	Tidak	RH	
R26	20	1	54	130	133	2	SMA	61,90%	Cukup	23	Rendah	71,40%	Tidak patuh	Tidak	RH	
R27	48	2	52	71	70	1	SD	61,90%	Cukup	23	Rendah	76%	Tidak patuh	Tidak	RH	
R28	54	1	65	81	50	1	SMA	61,67%	Cukup	21	Tinggi	100%	Patuh	Ya	RH	
R29	27	1	65	133	136	2	SMA	71,42%	Cukup	24	Rendah	67,85%	Tidak patuh	Ya	RH	
R30	54	1	58	206	231	2	SD	57,14%	Kurang	24	Rendah	79%	Tidak patuh	Ya	RH	
R31	50	1	50	67	66	1	SMP	61.90	Cukup	25	Tinggi	100	Patuh	Ya	RHZE	
R32	39	2	58	78	66	1	SMA	61.90	Cukup	25	Tinggi	100	Patuh	Tidak	RHZE	
R33	42	2	50	79	94	1	SMP	57.14	Kurang	25	Tinggi	100	Patuh	Tidak	RHZE	
R34	24	2	47	191	169	2	SMA	52.38	Kurang	21	Rendah	66.67	Tidak patuh	Tidak	RHZE	
R35	52	1	49	132	127	2	S MP	57.14	Kurang	21	Rendah	58.33	Tidak patuh	Ya	RH	
R36	40	2	40	183	128	2	SMA	47.62	Kurang	19	Rendah	63.06	Tidak patuh	Ya	RHZE	
R37	70	1	40	299	402	3	SD	47.62	Kurang	17	Rendah	16.67	Tidak patuh	Tidak	RHZE	
R38	31	2	50	76	62	1	SMP	61.90	Cukup	25	Tinggi	100	Patuh	Tidak	RHZE	
R39	29	1	45	59	85	1	SMA	61.90	Cukup	25	Tinggi	100	Patuh	Ya	RH	
R40	25	2	35	92	79	1	SMA	57.14	Kurang	25	Tinggi	100	Patuh	Tidak	RHZE	
R41	29	2	50	84	75	1	SMA	52.38	Kurang	21	Rendah	90.47	Patuh	Tidak	RHZE	

R42	57	1	45	160	144	2	SD	52.38	Kurang	21	Rendah	76.19	Tidak patuh	Tidak	RHZE	
R43	22	2	44	160	151	2	SMA	47.62	Kurang	19	Rendah	64.28	Tidak patuh	Tidak	RHZE	
R44	25	1	45	133	129	2	SMA	47.62	Kurang	21	Rendah	76.19	Tidak patuh	Ya	RHZE	
R45	21	2	44	145	135	2	SMA	52.38	Kurang	19	Rendah	16.67	Tidak patuh	Tidak	RH	
R46	36	1	45	133	129	2	SMP	52.38	Kurang	19	Rendah	78.57	Tidak patuh	Ya	RHZE	
R47	36	1	46	89	90	1	SMA	57.14	Kurang	21	Rendah	85.71	Patuh	Ya	RHZE	
R48	23	2	74	88	78	1	SMA	57.14	Kurang	23	Rendah	92.86	Patuh	Tidak	RHZE	
R49	24	2	35	77	76	1	SMA	52.38	Kurang	23	Rendah	78.57	Tidak patuh	Tidak	RHZE	
R50	50	2	63	181	167	2	SD	47.62	Kurang	19	Rendah	78.57	Tidak patuh	Tidak	RHZE	
R51	57	1	35	149	150	2	SD	47.62	Kurang	21	Rendah	85.71	Patuh	Tidak	RHZE	
R52	21	2	46	76	63	1	SMA	61.90	Cukup	25	Tinggi	100	Patuh	Tidak	RH	
R53	47	1	52	188	192	2	SMP	52.38	Kurang	21	Rendah	58.33	Tidak patuh	Tidak	RHZE	
R54	31	1	37	77	60	1	SMA	61.90	Cukup	25	Tinggi	100	Patuh	Ya	RHZE	
R55	46	1	44	180	194	2	SMP	47.62	Kurang	21	Rendah	78.57	Tidak patuh	Tidak	RHZE	
R56	21	1	44	95	70	1	SMA	47.62	Kurang	23	Rendah	78.57	Tidak patuh	Tidak	RHZE	
R57	38	1	85	184	194	2	SMP	52.38	Kurang	23	Rendah	16.67	Tidak patuh	Ya	RH	
R58	20	2	37	145	159	2	SMP	47.62	Kurang	19	Rendah	16.67	Tidak patuh	Tidak	RH	
R59	25	1	42	197	166	2	SMA	47.62	Kurang	21	Rendah	50	Tidak patuh	Ya	RH	
R60	69	1	49	66	84	1	SD	61.90	Cukup	25	Tinggi	100	Patuh	Tidak	RHZE	
R61	34	1	56	22	53	1	SMA	61,90	Cukup	25	Tinggi	100%	Patuh	Ya	RH	

R62	27	2	57	43	39	1	SMA	66,67	Cukup	25	Tinggi	100%	Patuh	Tidak	RH	
R63	58	1	45	44	41	1	SD	52,38	Kurang	25	Tinggi	90%	Patuh	Ya	RH	
R64	37	1	52,5	43	136	2	SMA	57,14	Kurang	23	Rendah	90%	Patuh	Ya	RH	
R65	26	2	50	31	17	1	SMA	76,19	Baik	25	Tinggi	100%	Patuh	Tidak	RH	
R66	21	1	45	35	22	1	SMA	61,90	Cukup	24	Rendah	100%	Patuh	Ya	RH	
R67	30	1	46,5	38	36	1	SMA	66,67	Cukup	25	Tinggi	100%	Patuh	Ya	RH	
R68	18	1	53,5	58	148	2	SMP	57,14	Kurang	24	Rendah	100%	Patuh	Tidak	RH	
R69	37	1	97	36	39	1	S1	85,71	Baik	25	Tinggi	100%	Patuh	Ya	RH	
R70	65	2	38	32	25	1	SD	66,67	Cukup	25	Tinggi	100%	Patuh	Tidak	RH	
R71	29	2	44,5	47	38	1	SMA	76,19	Baik	25	Tinggi	100%	Patuh	Tidak	RH	
R72	25	1	68	39	34	1	S1	80,95	Baik	25	Tinggi	100%	Patuh	Tidak	RH	
R73	40	1	47	44	30	1	SD	61,90	Cukup	25	Tinggi	100%	Patuh	Ya	RHZE	
R74	25	1	55	40	19	1	SMA	66,67	Cukup	25	Tinggi	100%	Patuh	Ya	RHZE	
R75	39	2	40	33	18	1	SMP	71,43	Cukup	25	Tinggi	100%	Patuh	Tidak	RH	
R76	19	1	59	35	10	1	SMA	61,90	Cukup	25	Tinggi	100%	Patuh	Tidak	RH	
R77	53	1	42	69	40	1	SMP	76,19	Baik	25	Tinggi	100%	Patuh	Ya	RH	
R78	47	2	36	132	37	2	SD	57,14	Kurang	25	Rendah	100%	Patuh	Tidak	RH	
R79	35	2	53	55	24	1	SMP	57,14	Kurang	25	Tinggi	100%	Patuh	Tidak	RH	
R80	31	1	90	80	50	1	S1	76,19	Baik	25	Tinggi	90%	Patuh	Tidak	RH	
R81	47	1	56	33	45	1	SMP	66,67	Cukup	25	Tinggi	100%	Patuh	Ya	RH	
R82	21	1	62	46	85	1	SMP	61,90	Cukup	25	Tinggi	100%	Patuh	Ya	RH	
R83	20	1	58,5	42	56	1	SMP	57,14	Kurang	24	Rendah	85%	Patuh	Tidak	RH	
R84	56	1	75,5	45	48	1	SMA	66,67	Cukup	25	Tinggi	100%	Patuh	Ya	RH	
R85	50	2	30	38	16	1	SD	66,67	Cukup	25	Tinggi	100%	Patuh	Tidak	RH	
R86	38	2	45,5	43	38	1	SMA	66,67	Cukup	25	Tinggi	100%	Patuh	Tidak	RH	
R87	71	1	43	38	51	1	SD	47,62	Kurang	25	Tinggi	100%	Patuh	Ya	RH	
R88	21	2	46	35	16	1	SMA	80,95	Baik	25	Tinggi	100%	Patuh	Tidak	RH	
R89	21	1	54	43	55	1	SMA	76,19	Baik	25	Tinggi	100%	Patuh	Ya	RH	
R90	41	1	65	56	66	1	SMA	71,43	Cukup	25	Tinggi	100%	Patuh	Ya	RH	

R91	26	1	43,5	108	84	2	SMP	61,90	Cukup	25	Tinggi	100%	Patuh	Ya	RH	

Questionnaire

FORM 2

Kuesioner Penelitian Model Peningkatan Pengetahuan dan Kepatuhan Pasien Tuberkulosis: Tinjauan Efek Samping Hepatotoksisitas.

A. Data Pasien

Inisial	:
Pengobatan bulan ke (pilih salah satu)	1 2 3 4 5 6 ……..
Umur, Tinggi badan, Berat badan	:
Jenis kelamin	:
Alamat	:
No Hp yang bisa dihubungi	:
Pendidikan akhir	:
Pekerjaan	:
Pendapatan (gaji) per bulan	:
Diagnosa penyakit	:
Komplikasi	:
Komorbiditas	:
Obat yang diminum	:

B. **Kepatuhan Pasien (MARS)**

Pilihlah pernyataan yang sesuai dengan keadaan saudara dengan memberikan tanda conteng (V)

Pertanyaan	Pilihan pernyataan				
	**Selalu**	**Sering**	**Kadang-kadang**	**Jarang**	**Tidak pernah**
Saya lupa minum obat					
Saya mengubah dosis minum obat					
Saya berhenti minum obat sementara					
Saya memutuskan untuk minum obat dengan dosis lebih kecil					

C. Pengetahuan pasien mengenai efek samping obat

Berikan centang (√) pada kotak di sebelah kanan yang anda pilih.

1. **Bagaimana anda tahu bahwa obat yang anda minum ini akan menyembuhkan anda?**

Obat ini akan mencegah and terjangkit penyakit TBC lagi- Ya ☐ Tidak ☐

Saya tidak paham kerja obat ini ☐

Jawaban lain:……………………………………………………………………………………….

2. Berapa jumlah obat TBC yang harus anda minum dalam sehari?

4 ☐

3 ditambah vitamin ☐

3 ☐

Tidak tahu ☐

Jawaban lain……………………………………………………………………………………….

Nama obat...

3. Berapa kali sehari anda harus minum obat TBC?

1 kali- ☐

2 kali- ☐

3 kali- ☐

Tidak tahu- ☐

Jawaban lain……………………………………………………………………………………….

4. Berapa lama anda harus minum obat TBC?

Lebih dari 9 bulan- Ya ☐ Tidak ☐

Kurang dari 6 bulan- Ya ☐ Tidak ☐

6-9bulan- Ya ☐ Tidak ☐

Tidak tahu- ☐

Jawaban lain……………………………………………………………………………………….

5. Apa efek samping obat TB yang anda ketahui?

Gangguan hati	Ya ☐ Tidak ☐
Mual dan muntah	Ya ☐ Tidak ☐
Nyeri perut	Ya ☐ Tidak ☐
Kulit dan kelopak mata kuning	Ya ☐ Tidak ☐
Gangguan syaraf	Ya ☐ Tidak ☐
Rasa terbakar, gringgingen/semuten di jari tangan/kaki-	Ya ☐ Tidak ☐
Demam	Ya ☐ Tidak ☐
Gatal-gatal	Ya ☐ Tidak ☐

Jawaban lain…………………………………………………….

6. Apa yang sebaiknya anda lakukan jika mengalami efek samping obat TBC?

Obat dihentikan-	Ya ☐ Tidak ☐
Konsultasi dokter-	Ya ☐ Tidak ☐
Pemeriksaan darah ke laboratorium-	Ya ☐ Tidak ☐
Tidak tahu-	☐

Jawaban lain……………………………………………………………………………………….

Efek samping apa yang anda alami selama mengkonsumsi obat TBC?

Gangguan hati	Ya ☐ Tidak ☐
Mual dan muntah	Ya ☐ Tidak ☐

Nyeri perut Ya ☐ Tidak ☐

Kulit dan kelopak mata kuning Ya ☐ Tidak ☐

Gangguan syaraf Ya ☐ Tidak ☐

Rasa terbakar, gringgingen/semuten di jari tangan/kaki- Ya ☐ Tidak ☐

Demam Ya ☐ Tidak ☐

Gatal-gatal Ya ☐ Tidak ☐

Jawaban lain……………………………………………………….

7.

Terima kasih telah berpartisipasi dalam penelitian ini, Semoga kondisi Anda segera membaik
